# The Moral Side of Dentistry

**Published:** 1901-09-15

**Authors:** Geo. W. Clapp

**Affiliations:** Cygnet, Ohio


THE
### DENTAL REGISTER.

**Vol. LV. September 15, 1901. No. 9.**

### THE MORAL SIDE OF DENTISTRY.

(Second Paper.)

BY GEO. W. CLAPP, D.D.S., CYGNET, OHIO.

*The Influence of the Colleges.*—If a competent dentist be asked his opinion of our leading dental colleges, his reply will usually be very favorable. The substance of it would probably be that they are founded on wise principles to meet an urgent need; that the men in charge are usually competent and progressive, and that the colleges form our greatest legacy from the past and richest hope for the future. And aside from any merely personal preference, he would probably say that in any of them the young man who seeks to do so may learn all a college can teach him concerning dentistry.

But when we turn from the intellectual and technical training and seek information concerning the moral influences which our colleges exercise on the aspirant for professional honors, this unanimity of favorable opinion disappears, and, save from those engaged in college work, the reply is usually a smile of amusement, followed by the statement that if the colleges exercise any disciplinary moral influence on those students who keep their work up, it is usually in such small degree and of so mild a character that the recipient is entirely unconscious of it.

The reason for such a condition is not far to seek. It is not because the men in charge of our colleges are not good men, but because so many practitioners do not hold that high estimate of the moral side of dentistry which must precede the attainment of its desired position in the eyes of the public. The men in charge of our colleges are, for the most part, moral men; few are more highly honored among us, and their influence during their brief contact with the student doubtless makes for good. They have great influence in shaping the intellectual life of the profession, but one fact which seems not to be fully appreciated prevents, to a great extent, their shaping its moral life. That fact is that up to the present the profession, as constituted by the practitioners, has required nothing of the private morals of its members. It is not therefore in a position to dictate to the schools what their requirements shall be, or to exact from the aspirant for professional fellowship even the slightest private moral rectitude. It might be thought that though the profession, as a body, is thus impotent, the colleges would be keenly alive to their duties, and would take adequate steps for the discharge of one of their greatest responsibilities. In a future paper it will be shown that they have not, so far as any noteworthy, practical results are concerned. If a student's character can be made to stand a very superficial examination its inner corruption need not hinder his getting through school, and when his diploma is once in his hand there are none to whom he must answer; and, criminal practices aside, he may order his private and professional life according to the inmost desires of his heart, however base that may be.

The result of this lack of requirement on the part of the profession and the colleges is, that it is possible for a youth of a very bad moral character to graduate from some, if not any, of our best colleges. At this moment there is in the senior class of one of the best colleges in the country a youth who is a frequent drinker, a gambler and a cheat. One of his vacations was spent, with other members of the same class, within the confines of a jail as the result of a drunken carouse.

During his student days he has been matriculated in two of the leading dental colleges, and, ere this appears in print, will probably be graduated from one of them and endorsed to the public by a diploma signed by men of world-wide fame. As I write these lines there lie before me letters from both faculties before whom this young man pursued his studies. To both faculties was addressed the question, "Do you suspend or expel a student for known affliction with specific diseases, alcoholism, licentiousness or other moral aberration?" The secretary of one faculty replies, "We have had no such cases called to our attention;" and the secretary of the other faculty writes, "Five students have been expelled for specific immoralities during the past three years;" and this fellow, who has been drunk at least once a week during every term, was not one of the five. If these faculties did not know this student's character they were the only persons in any way connected with the schools who did not, and they might have learned. It would be possible to multiply such cases in lesser degree from accurate sources, but this case will do for the present.

To the further question, "If you have reason to suppose that his (the student's) future career will not be creditable to the profession, what course do you pursue?" both secretaries reply in effect that they would dismiss him. Why didn't they dismiss this man, then? What credit can such a moral leper be to any profession? Every breath that blows across him is laden with the rottenness of moral putrefaction; his very touch is contamination, and his continued presence is a moral pestilence. What fitness has he for any trust; what fitness to be granted professional rank and title; what fitness to be received into the fellowship of men who are seeking to place their calling upon a high and honorable plane in the eyes of all people? When he has been graduated, to what professional discipline can he be made amenable? What can such a one care about the Code of Ethics? In the eyes of the public he will be a dentist, graduated by such a school, with a diploma bearing names known wherever dentistry is known. Is it surprising that because of his actions the profession, the school and the men whose names are on his diploma will be lowered, perhaps forever, in the eyes of those who finally find him out?

A search for the reason why the faculties did not dismiss the young man referred to reveals several facts of the utmost importance to the profession at large, and having profound influence on the future. They are as follows: First, that there is no searching moral discipline in our schools; second, that the fault for such a condition rests partly on the faculties and partly upon dentists generally; third, that there is small hope of wholly removing such conditions until dentists generally appreciate the influence of private morality on professional standing and hold the schools accountable for the men they endorse.

Take each statement separately. First. There is no searching moral discipline in our schools. While the writer has personal acquaintance with only a few of our leading schools, he has sought and gained opportunities for conversation with credible students of half a dozen of the best colleges in the land. Of these schools at least, and they are doubtless as strict as any, it is possible to state definitely that the moral side of the profession, so far as it relates to the private morals of the students, receives no systematic official emphasis. There is no feeling that the profession upon which they are to enter requires of them anything more than professional skill, or that it has a right to inquire into their private lives and insist that they be of such cleanliness as will make the men worthy of confidence in positions of trust. The result is that there are among the students of the best colleges victims of specific diseases, gamblers, habitual drinkers, cigarette fiends, and were not the sources of information confidential, it would not be impossible to specify schools and individuals.

It is true that in the best schools a case of flagrant immorality can sometimes be punished by expulsion, if prosecuted vigorously, but there is a feeling that the private morals of the students are not subjects of investigation by the faculties, and that, unless complaints are lodged, there is no need to inquire what manner of men they, by name, are to endorse to the public.

Second. The fault for this condition of affairs rests partly upon the schools and partly upon the profession generally. It rests upon the schools because it would be possible, if they would take the trouble, to select for students such as are worthy. It is as possible to find out what a man is as what he knows. When it is possible to select a man to keep books, count money, tend a switch, or run a train, and know beforehand what sort of character he will bring to the work, it is preposterous to claim that the character of the man who is to perform operations and prescribe treatments may not be as thoroughly known.

As we sift the matter more finely it is seen that the men in charge of our colleges have either not appreciated their moral responsibilities, or have been woefully negligent. On the intellectual and technical sides the responsibilities are better appreciated and more fully met, but it needs to be graven on the mind of every dental faculty in the land that the moral responsibilities are not less great than the technical, and that they are too often slightingly discharged.

The schools stand between the mass of applicants, fit and unfit, and the fields of professional achievement and honor. To the unfit man they should be an insuperable barrier past which neither money, nor favor, nor persistence, could carry him ; to the fit man they should be a professional mother. The first great function of the school should be that of selector. It should, without fear or favor, weed the unfit from among the fit, and admit to its training only those who, after careful scrutiny, prove worthy. And it should be a cardinal principle of such weeding that nothing can excuse or compensate for moral baseness ; that whatever other qualifications the grossly immoral man may have, he is a social leper, unclean, infectious, loathsome. If our faculties were as interested and intelligent concerning moral selection as they are regarding mental selection, many students who now hinder and degrade the profession would find admission impossible. In the next paper an attempt will be made to show what some of our leading schools are requiring in the way of moral fitness and to outline a plan whereby a man's character may be known, at least much better than by any plan now in practical operation.

The blame comes more closely home to the men in charge of the schools, because they are the ones to whom the question as to who is worthy to be admitted to the profession has largely been left. The dentist busy in his office, the State board meeting quarterly, the man suffering dental pain, and other such, can not pause to carefully investigate the character of the new graduate. If he carry the diploma of a reputable school it is supposed to show that the men in charge of that school have looked him up, have found him worthy, have carefully trained him and finally have signed a statement of his fitness. So far as this is not the case, the men who sign the diploma are not true to themselves, the profession and the public.

The blame which rests on dentists in general is antecedent to this fault of the schools, and in large measure responsible for it. It is, as has been said, that the profession requires little or nothing in the way of private morals of its members, and therefore it can not dictate to the faculties or enforce moral discipline on the graduate.

Third. There is small hope of wholly altering such conditions until dentists generally appreciate the influence of private morality on professional standing, and hold the schools accountable for the men they endorse. We can hardly insist that the faculties maintain a higher standard than we are willing to support by our own private conduct, or expect them to make operative a standard which our lives daily degrade and belie in the eyes of the under-graduate. The mere dictum of any man or body of men is not sufficient to establish such a reform. Back of the word must come a strong endorsement by the life; that alone can give the word power. If we are to insist that the students who are to be distinguished by the diplomas of our good schools must be free from diseases of vice, we, as a body of dentists, must be free from such diseases. If the students are to be free from the excessive use of intoxicants, we must first free ourselves. If they are to be men worthy of confidence in positions of trust, we also must be such. Not until we are free from the evils we prohibit in them can we hope for any widespread improvement.

If the time ever comes when the line of demarcation between the morally living and the morally dead is as sharply drawn as it is between the physically living and the physically dead, and should the morally dead, like the physically dead, be thrown out of the system, much will have been done toward the correction of many present evils. And he who can so invigorate the profession that it shall hasten to establish such reparative processes will do it one of the greatest kindnesses that any loving adherent can render.

